# Preliminary report of a clinical trial supporting the sequential use of an attenuated E39 peptide (E39') to optimize the immunologic response to the FBP (E39+GM-CSF) vaccine

**DOI:** 10.1186/2051-1426-3-S2-P156

**Published:** 2015-11-04

**Authors:** Doreen Jackson, Na Qiao, Julia M Greene, Diane Hale, John Berry, Alfred Trappey, Timothy Vreeland, Guy Clifton, Nuhad Ibrahim, Annie Toms, George E Peoples, Elizabeth A Mittendorf

**Affiliations:** 1 San Antonio Military Medical Center, Converse, TX, USA; 2 The University of Texas MD Anderson Cancer Center, Houston, TX, USA; 3 San Antonio Military Medical Center, San Antonio, TX, USA; 4 MD Anderson Cancer Center, Houston, TX, USA; 5 Cancer Vaccine Development Program, San Antonio, TX, USA

## Background

Folate Binding Protein (FBP) is overexpressed in breast, endometrial, and ovarian cancers. E39 (FBP191-199, EIWTHSYKV) is an HLA-A2 restricted FBP peptide vaccine already shown to generate significant *in vivo* immunologic response (IR) in a Phase I/IIa trial in endometrial and ovarian cancer. We are investigating a novel vaccination series using E39 and E39' (EIWTFSTKV), an attenuated version of E39, in a Phase Ib, randomized, single-center trial (NCT020196524) evaluating IR and monitoring long-term immunity. We present the initial IR analysis to the primary vaccination series (PVS).

## Methods

HLA-A2+ breast or ovarian cancer patients were enrolled after completion of standard of care therapy and randomized into three arms: EE-6 inoculations of E39; EE'-3 inoculations of E39 then 3 of E39'; or E'E-3 inoculations of E39' then 3 of E39. PVS includes 6 inoculations total, one every 3-4 weeks containing 250mcg GM-CSF + 500mcg peptide in the first 5 patients per arm and 1000mcg of peptide in second 5 patients. To assess the *in vivo* IR, local reaction(LR) was measured 48hours after each inoculation (R1-6), and delayed type hypersensitivity (DTH) was measured pre-vaccine(R0), 1 and 6-months after the PVS (RC1, RC6). E*x vivo* IR was measured via dextramer assay for E39-specific CD8+ T-cells at R0, RC1, and RC6. Statistical analyses included descriptive statistics, t-test, Chi-squared, Fisher's exact test and ANOVA as appropriate.

## Results

Thirty-two patients were enrolled (EE n=10, EE' n=11, E'E n=11), with no clinicopathologic differences between groups, or significant toxicities appreciated. *In vivo* LR showed a significant difference within EE and EE' arms. LR peaked in EE at R4, while EE' continued to increase in size throughout the PVS(EE p=0.04, EE' p=0.02, E'E p=0.74). An increase in *in vivo* DTH was observed within arm EE' from R0-RC1-RC6 (EE p=0.72, EE' p < 0.05, E'E p=0.41). Ex vivo analysis of IR revealed no significant difference between groups (p=0.39), nor within groups (EE p=0.82, EE' p=0.58, E'E p=0.49).

## Conclusion

In this Phase Ib trial comparing three vaccination strategies in ovarian and breast cancer patients, preliminary analysis revealed E39, given sequentially with or without E39', is immunogenic. The *in vivo* response is enhanced with the use of the attenuated E39' after E39. This was observed in the EE' arm, this vaccination sequence producing the most prominent LR and DTH responses. Continued analysis of immunologic responses as more data is obtained will further elucidate the optimal vaccination series for the prevention of recurrence in breast and ovarian cancer.

## Trial registration

ClinicalTrials.gov identifier NCT020196524.

